# Cellular MRI Reveals Altered Brain Arrest of Genetically Engineered Metastatic Breast Cancer Cells

**DOI:** 10.1155/2019/6501231

**Published:** 2019-01-08

**Authors:** Katie M. Parkins, Amanda M. Hamilton, Veronica P. Dubois, Suzanne M. Wong, Paula J. Foster, John A. Ronald

**Affiliations:** ^1^Robarts Research Institute, The University of Western Ontario, London, Ontario, Canada; ^2^The Department of Medical Biophysics, The University of Western Ontario, London, Ontario, Canada; ^3^Lawson Health Research Institute, London, Ontario, Canada

## Abstract

**Purpose:**

The combined use of anatomical magnetic resonance imaging (MRI), cellular MRI, and bioluminescence imaging (BLI) allows for sensitive and improved monitoring of brain metastasis in preclinical cancer models. By using these complementary technologies, we can acquire measurements of viable single cell arrest in the brain after systemic administration, the clearance and/or retention of these cells thereafter, the growth into overt tumours, and quantification of tumour volume and relative cancer cell viability over time. While BLI is very useful in measuring cell viability, some considerations have been reported using cells engineered with luciferase such as increased tumour volume variation, changes in pattern of metastatic disease, and inhibition of *in vivo* tumour growth.

**Procedures:**

Here, we apply cellular and anatomical MRI to evaluate *in vivo* growth differences between iron oxide labeled naïve (4T1BR5) and luciferase-expressing (4T1BR5-FLuc-GFP) murine brain-seeking breast cancer cells. Balb/C mice received an intracardiac injection of 20,000 cells and were imaged with MRI on days 0 and 14. Mice that received 4T1BR5-FLuc-GFP cells were also imaged with BLI on days 0 and 14.

**Results:**

The number of signal voids in the brain (representing iron-labeled cancer cells) on day 0 was significantly higher in mice receiving 4T1BR5 cells compared to mice receiving 4T1BR5-FLuc-GFP cells (*p* < 0.0001). Mice that received 4T1BR5 cells also had significantly higher total brain tumour burden and number of brain metastases than mice that received 4T1BR5-FLuc-GFP cells (*p* < 0.0001).

**Conclusions:**

By employing highly sensitive cellular MRI tools, we demonstrate that engineered cells did not form tumours as well as their naïve counterparts, which appear to primarily be due to a reduction in cell arrest. These results indicate that engineering cancer cells with reporter genes may alter their tropism towards particular organs and highlight another important consideration for research groups that use reporter gene imaging to track metastatic cancer cell fate *in vivo*.

## 1. Introduction

The ability to accurately quantify tumour growth in preclinical cancer models is critical for effective study of tumour biology, metastatic spread, and treatment response. While some subcutaneous and orthotopic tumour volumes can be measured using either manual or electronic calipers, this relies on a fairly developed-palpable tumour. For instance, many murine cancer models have been developed to try to improve the clinical relevance such that micro- or macrometastases form in the brain, bone, and lung and thus are not measurable with calipers. To better monitor the longitudinal growth of subpalpable or metastatic disease relies on one employing noninvasive imaging techniques.

There are a number of cellular and molecular imaging modalities that can be used to noninvasively measure tumour size, location, metabolism, and metastatic burden in preclinical cancer models such as ultrasound (US), computed tomography (CT), magnetic resonance imaging (MRI), single-photon emission computed tomography (SPECT), positron emission tomography (PET), and optical imaging modalities such as fluorescence imaging and bioluminescence imaging (BLI) [1–3]. Among these, BLI continues to be one of the most employed technologies for evaluating tumour growth and viability over time due to its high sensitivity, high throughput nature, and relative cost-effectiveness. For BLI, tumour cell lines are engineered to express a luciferase reporter gene, most commonly firefly luciferase (FLuc), which produces light as a product of oxidation of a matching luciferin substrate. The relative amount of light produced at a particular location can provide indirect measures of total cancer cell viability over time [4]. This is an important consideration as larger tumours may have edema and/or necrosis, contributing to imaging measures of tumour volume, and as a result, overestimate the number of viable cancer cells present [5]. Furthermore, in models evaluating treatment response, the amount of viable tissue within the tumour may change before any anatomical changes occur. Thus, BLI can provide valuable complementary information to measures of tumour size with either calipers or anatomical imaging modalities such as MRI.

We have previously demonstrated the combined use of BLI, anatomical MRI, and cellular MRI tools for monitoring experimental breast cancer brain metastasis in mice [6]. Cellular MRI requires cancer cells to be labeled with superparamagnetic iron oxide (SPIO) nanoparticles in culture prior to transplantation into mice [7]. The iron causes a distortion in the magnetic field, leading to a loss of signal in an iron-sensitive MRI sequence. The blooming artifact produced by the SPIO is larger than the cell itself, and as a result, we can visualize single cancer cells arresting in the brain at the time of injection as well as nondividing cancer cells that retain their iron label over time [8]. A small portion of the cancer cells will divide and lose their iron label but can be visualized with conventional MRI. By using cellular MRI, anatomical MRI, and BLI simultaneously in the same animals, we can acquire measurements of viable single cell arrest and cell clearance as well as follow the growth and/or changes in viability of tumours in the brain over time [6].

While BLI is very useful in evaluating the fate of many different cell populations *in vivo*, including cancer cells, in recent years some considerations for the use of BLI have been reported. Following the engineering of cells with luciferase, several studies have noted changes in tumour volume variation across animals, altered cancer cell tropism toward particular organs, as well as differences in tumour growth rates [9–12]. The ability to sensitively track the dissemination, arrest, dormancy, or growth of single cells with cellular MRI may provide new insights into potential effects of cell engineering. The objective of this work was to use cellular and anatomical MRI to characterize the *in vivo* growth patterns of naïve and lentiviral-engineered brain-seeking triple negative breast cancer cells coexpressing fluorescent and bioluminescent reporters in the mouse brain.

## 2. Materials and Methods

### 2.1. In Vitro Studies

#### 2.1.1. Cell Engineering

Brain-seeking mouse mammary carcinoma cells (4T1BR5) were a kind gift from Dr. Patricia Steeg's lab (NIH, Centre for Cancer Research) and engineered to stably coexpress red-shifted *Luciola italica* luciferase (FLuc) and GFP using a commercial lentiviral vector (RediFect Red-FLuc-GFP lentiviral particles; PerkinElmer, USA). Cells were transduced at a multiplicity of infection of 20 and sorted based on GFP expression using a FACSAria III flow cytometric cell sorter (BD Biosciences, San Jose, CA, USA). The resultant 4T1BR5-FLuc-GFP cells were maintained in DMEM containing 10% FBS at 37°C and 5% CO_2_. All *in vitro* experiments were performed in triplicate.

#### 2.1.2. Iron Labeling

For iron labeling, 2 × 10^6^ cells were plated in a 75 cm^3^ flask, supplemented with DMEM containing 10% FBS, and allowed to adhere for 24 hours. Cells were incubated for an additional 24 hours with 10 mL media containing 25 *μ*g/mL of MPIO beads (0.9 *µ*m in diameter, 63% magnetite, labeled with Flash Red; Bangs Laboratory, Fishers, IN, USA). Cells were washed three times with Hanks balanced salt solution (HBSS) and then trypsinized with 0.25% Trypsin-EDTA. The cells were then collected and thoroughly washed three more times with HBSS to remove unincorporated MPIO before cell injection and *in vitro* evaluation.

#### 2.1.3. Propidium Iodide Cell Cycle Assay

Breast cancer cells (naïve and engineered 4T1BR5) were cultured as stated above. Cells were centrifuged at 1000 rpm for 5 minutes. Cell pellets were then fixed with 500 *μ*l of 70% ethanol for 30 minutes in 4°C, washed twice with phosphate-buffered saline (PBS), and centrifuged at 850 g. Cells were then treated with 50 *μ*L of RNase (100 *μ*g/mL). The mixture was kept in a water bath at 37°C for 30 minutes prior to staining with 200 *μ*L of propidium iodide solution (50 *μ*g/mL) and then analyzed by flow cytometry using a FACSAria III flow cytometric cell sorter (BD Biosciences, San Jose, CA, USA).

#### 2.1.4. Proliferation Assay

Vybrant MTT (3-(4,5-dimethylthiazolyl-2)-2,5-diphenyltetrazolium bromide) proliferation assays were used to evaluate whether genetic engineering had an effect on *in vitro* proliferation. 4T1BR5 and 4T1BR5-FLuc-GFP cells were seeded in 96-well plates (2.0  ×  10^3^ cells per well) with 0.25 mL of media, and cell proliferation was evaluated at 0, 24, 48, and 96 hours. MTT solution (20 *µ*L) was added to each well, and absorbance at 450 nm was measured using a microplate spectrophotometer (Fluoroskan Ascent FL, ThermoLabSystems).

#### 2.1.5. Clonogenic Assay

Naïve and engineered 4T1BR5 cells were seeded in 6-well plates (1.0 × 10^3^ cells per well) with 2 mL of media. The number of colonies in each well was manually counted using a hemocytometer at 72 hours after plating.

### 2.2. In Vivo Studies

#### 2.2.1. Experimental Breast Cancer Brain Metastasis Model

Animals were cared for in accordance with the standards of the Canadian Council on Animal Care and under an approved protocol of the University of Western Ontario's Council on Animal Care (protocol number: 2014-026). To deliver MPIO-labeled 4T1BR5 or 4T1BR5-FLuc-GFP cells into the brain, 2.0 × 10^4^ cells were injected into the left ventricle of female BALB/c mice (*n*=16; 6–7 weeks old; Charles River Laboratories, Wilmington, MA, USA). Cells were suspended in 0.1 mL of HBSS, and image-guided injections into the left ventricle were performed using a Vevo 2100 ultrasound system (FUJIFILM VisualSonics Inc., Toronto, ON, Canada). MRI was performed on all sixteen mice on days 0 and 14 after intracardiac injection. In addition, mice that received 4T1BR5-FLuc-GFP cells had BLI performed on days 0 and 14 after intracardiac injection.

#### 2.2.2. MRI

All MRI scans were performed on a 3T GE clinical MR scanner (General Electric) using a custom-built gradient coil and a custom-built solenoidal mouse brain radiofrequency coil [7, 13]. Mice were anesthetized with isoflurane (2% in 100% oxygen), and images were obtained using a 3D balanced steady-state free precession (bSSFP) imaging sequence (Fast Imaging Employing Steady-State Acquisition (FIESTA) on the GE system) which has been previously optimized for iron detection [14]. The scan parameters for day 0 images were repetition time (TR) = 8 ms, echo time (TE) = 4 ms, bandwidth (BW) = 41.7 kHz, flip angle (FA) = 35 degrees, averages (NEX) = 2, phase cycles = 4, and matrix = 150 × 150. Total scan time was 15 minutes per mouse. For day 14 images, a longer scan time was required for tumour detection, and so imaging parameters were TR = 10 ms, TE = 5 ms, BW = 12.5 kHz, FA = 35 degrees, NEX = 2, phase cycles = 8, and matrix = 150 × 150. Total scan time was 35 minutes per mouse.

#### 2.2.3. BLI

BLI was performed using a hybrid optical/X-ray scanner (IVIS Lumina XRMS In Vivo Imaging System, PerkinElmer). Mice were anesthetized with isofluorane (2% in 100% oxygen) and received a 150 *μ*L intraperitoneal injection of D-luciferin (30 mg/mL; Syd Labs, Inc., MA, USA), and BLI images were captured for up to 35 minutes after injection.

#### 2.2.4. Image Analysis

MRI images were analyzed using OsiriX software (Pixmeo, SARL, Bernex, Switzerland). Day 0 images were analyzed by manually counting signal voids (representing iron-labeled cells) in every slice throughout the whole brain. For day 14 images, brain metastases were manually traced and 3D tumour volumes were reconstructed using the OsiriX volume algorithm. *In vitro* BLI signal was measured with region-of-interest (ROI) analysis using Living Image Software (PerkinElmer). An ROI was drawn around each well, and average radiance (photons/second/cm^2^/steradian) was measured.

#### 2.2.5. Histology

At endpoint, mice were sacrificed by pentobarbital overdose and perfusion fixed with 4% paraformaldehyde. Mouse brains were removed and cryopreserved in ascending concentrations of sucrose (10, 20, and 30% w/v) in distilled water for at least 1 hour each. Brains were immersed in the optimal cutting temperature (OCT) compound, oriented in a sectioning plane parallel to that of MRI, and frozen using liquid nitrogen. Frozen sections (10 *μ*m) were collected and stained using hematoxylin and eosin (H&E) to visualize tumour morphology.

#### 2.2.6. Statistics

A power analysis was performed using *G*∗Power software to determine the appropriate sample size for this study. All statistics were calculated using GraphPad Prism 4. A Student's two-tailed unpaired *t*-test was used to compare conditions in *in vitro* experiments as well as between animal groups. A nominal *p* value less than 0.05 was considered statistically significant.

## 3. Results

**Figure 1:**
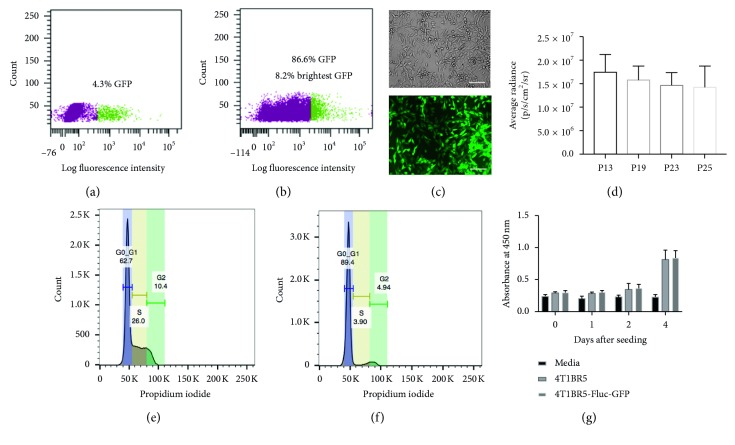
In vitro characterization of engineered 4T1BR5 cell line: after lentiviral transduction, 4.3% of the total population was GFP positive (purple = GFP negative; green = GFP positive) (sort 1) (a). These cells were grown out and found to be 86.6% GFP positive during the second sort. From this population, we sorted out the brightest 8.2% of cells (purple = cells not collected; green = 8.2% brightest GFP expression that were collected) (sort 2) (b). Brightfield and GFP expression of resultant 4T1BR5-Fluc-GFP cells (scale bar = 100 microns) (c). Luciferase activity of 4T1BR5-Fluc-GFP cells over multiple passages (P13–P25) in culture (d). Cell cycle arrest analysis of 4T1BR5 (e) and 4T1BR5-Fluc-GFP cells (f). Cellular proliferation of naïve 4T1BR5 and 4T1BR5-FLuc-GFP cells (g). Data are presented as mean ± SEM.

After lentiviral transduction, 4.3% of the total population was found to be GFP positive, and these cells were sorted, expanded, and then sorted a second time (Figure 1(a)). During the second sort, we found 86.6% of the cells to be GFP positive. From this population, we isolated the brightest GFP cells (8.2%) by FACS and expanded them in culture to obtain a population of 4T1BR5-FLuc-GFP cells that were near 100% GFP positive (Figure 1(b)). *In vitro* GFP expression was assessed using fluorescence microscopy (Figure 1(c)) and FLuc with BLI (Supplementary Figure 1(a)). To determine the stability of our reporter genes, we performed *in vitro* BLI of 4T1BR5-FLuc-GFP cells and found no significant differences in luciferase activity over multiple passages in culture (Figure 1(d)). Similarly, using FACS, we found no significant differences in mean GFP signal intensity over 10 cell passages (Supplementary Figure 1(c)). Next, we determined whether cell cycle differences existed between naïve and engineered cells by performing a propidium iodide cell cycle arrest assay. As displayed in Figure 1(f), we observed a decrease in the number of cells in the S phase and an increase in the number of cells in *G*_0_/*G*_1_ for engineered 4T1BR5 cells compared to naïve cells (Figure 1(e)). We evaluated differences in cellular proliferation between naïve 4T1BR5 and 4T1BR5-FLuc-GFP cells over a four-day period using an MTT assay and found there were no significant differences in cell growth at any of the time points (Figure 1(g)). We also performed a clonogenic assay to evaluate differences in the ability of each cell population to form colonies and found there was no significant difference in the number of colonies formed between naïve and engineered 4T1BR5 cells (Supplementary Figure 1(b)).

**Figure 2:**
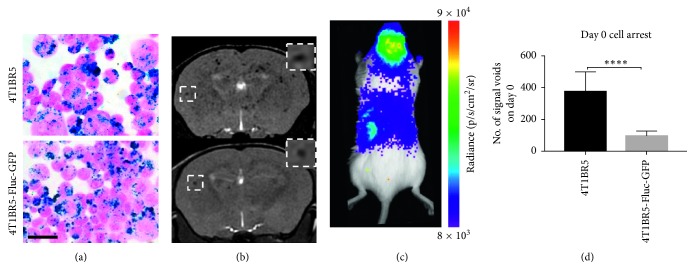
Altered initial brain arrest of engineered 4T1BR5 cells as detected with iron oxide cellular MRI: Perl's Prussian blue staining identifies iron-labeled cells in blue (scale bar = 50 microns) (a). Iron-labeled cells were visualized in brain MR images as discrete signal voids on day 0 (*n*=8 per group); insets showing example of distinct voids (b). Brain and body BLI signal was also detectable on day 0 in mice that received luciferase-expressing cells (*n*=8) (c). The number of voids (in MR images) on day 0 in mice receiving naïve 4T1BR5 cells or 4T1BR5-Fluc-GFP cells (d). The data are presented as mean ± SEM.

MRI and BLI data from the day of intracardiac injection (day 0) are shown in Figure 2. Perl's Prussian blue stain was performed to show both 4T1BR5 and 4T1BR5-FLuc-GFP cells were efficiently (>90%) labeled with MPIO prior to intracardiac injection (Figure 2(a)). Iron-labeled cells were visualized in MR images as discrete signal voids distributed throughout the mouse brain (Figure 2(b)). BLI signal was detected in the brain and body of mice that received 4T1BR5-FLuc-GFP cells on day 0 (Figure 2(c)). Importantly, the number of discrete signal voids in the brain on day 0 was significantly higher in mice that received 4T1BR5 cells (379 ± 42 voids) than 4T1BR5-FLuc-GFP cells (98 ± 10 voids; *p* < 0.0001), despite mice receiving equivalent numbers of cells intracardially (Figure 2(d)).

**Figure 3:**
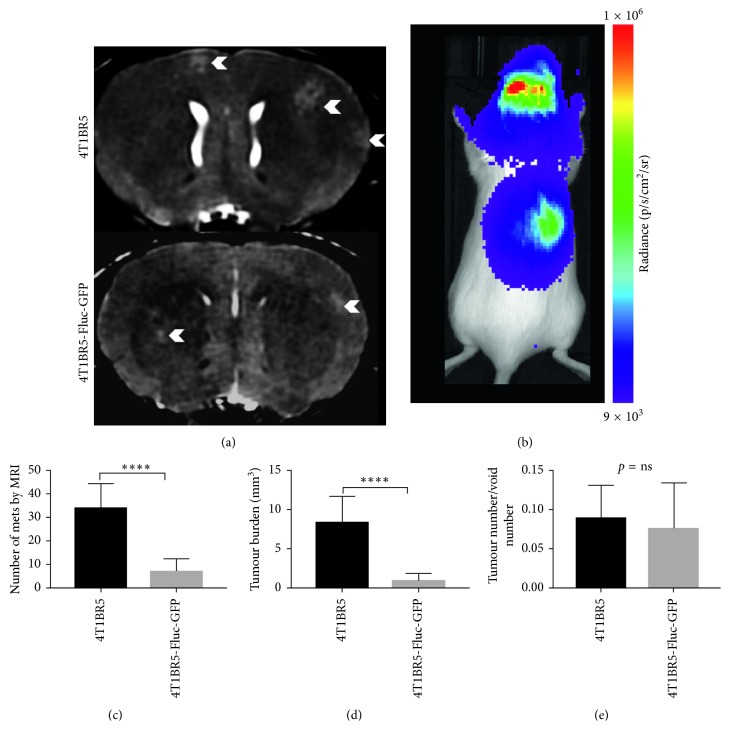
Differences in endpoint metastatic burden between naïve and engineered 4T1BR5 cells: representative MR slices from each group, and brain metastases indicated by white arrowheads (*n*=8 per group) (a). BLI signal was detected in the brain and body of mice that received luciferase-expressing cells (*n*=8) (b). The number of brain metastases (c) and total brain tumour burden (d) in mice that received naïve and luciferase-expressing 4T1BR5 cells. The ratio of the number of tumours at endpoint over the number of signal voids on day 0 was also compared between groups (e). The data are presented as mean ± SEM.

**Figure 4:**
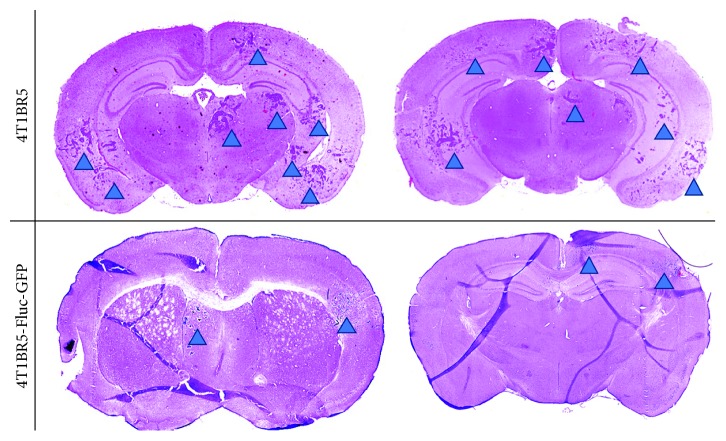
Histological differences in tumour burden between naïve and engineered 4T1BR5 cells: the presence of tumours was confirmed using hematoxylin and eosin (H&E) staining. Tumours are indicated with blue arrowheads.

On day 14, brain metastases appeared as hyperintense regions in MR images.  Figure 3(a) shows an MR slice from a representative mouse brain from each group with white arrowheads pointing to metastases. All mice in this study, regardless of the cell line injected (naïve or engineered), had MR detectable metastases at endpoint.  Figure 3(b) shows a whole body BLI image from a mouse with 4T1BR5-FLuc-GFP tumours. All mice that received 4T1BR5-FLuc-GFP cells had BLI detectable metastases in both the brain and other parts of the body. In day 14 MR images, tumours were manually counted throughout the entire mouse brain. In addition, tumour boundaries were manually traced, and 3D tumour volumes were determined using the OsiriX volume algorithm. MR image analysis revealed that mice that received 4T1BR5 cells had a significantly higher number of brain metastases (34 ± 4 tumours) than mice that received 4T1BR5-FLuc-GFP cells (7 ± 2 tumours; *p* < 0.0001) (Figure 3(c)). We also found that mice that received 4T1BR5 cells had significantly more total brain tumour volume (8.27 ± 1.15 mm^3^) than mice who received the 4T1BR5-FLuc-GFP cells (1.03 ± 0.28 mm^3^; *p* < 0.0001) (Figure 3(d)). We also evaluated whether the relative number of tumours that formed between the groups was related to the initial number of cells seeding the brain. To do so, we evaluated the ratio of the tumour number at endpoint to the initial number of voids on day 0 and found there were no significant differences between the two mouse cohorts (Figure 3(e)). Finally, the presence of tumours was also confirmed using hematoxylin and eosin (H&E) staining. Qualitatively, mice that received 4T1BR5 cells had more metastases in the brain compared to mice with 4T1B3R5-FLuc-GFP tumours (Figure 4).

## 4. Discussion

In this study, we used anatomical and cellular MRI to characterize *in vivo* arrest of single cancer cells and growth differences in an established preclinical model of brain metastasis between naïve triple negative breast cancer cells and cells engineered to stably express fluorescence and bioluminescence reporter genes. Here, we report mice that received naïve 4T1BR5 cells had significantly more brain metastases and significantly higher total brain tumour burden than mice that received engineered 4T1BR5-FLuc-GFP cells. Furthermore, through the use of iron-based cellular MRI, we were able to determine that mice that received the naïve cell line also had significantly more discrete signal voids (representing iron-labeled cells) in the brain on the day of intracardiac injection compared to mice that received our engineered cell line. This highlights that differences in metastatic tumour burden between engineered and naïve cells may not only be due to differences in *in vivo* growth rates of engineered cells as suggested by others [9–12] but may also arise very early in the metastatic process by altering organ seeding efficiency.

An important step in the metastatic cascade is the initial arrest of circulating tumour cells. Our results point to decreased arrest of engineered cancer cells being the probable explanation for differences in endpoint tumour number and burden. In the current study, we found the engineering of the 4T1BR5 cell line caused an increase in the number of cells in *G*_0_/*G*_1_ *in vitro*. Altered cell arrest could be the result of several possibilities including selection of a subset of cells with reduced arrest ability, the use of lentiviral vectors that integrate into the genome causing altered expression of genes important for homing and arrest, or an early immune response to a specific transgene (e.g., GFP or luciferase). Baklaushev et al. suggested an early immune response to luciferase may limit the spread of metastatic cells when they migrate as single cells in the vasculature. Since immune competent mouse models and reporter gene imaging are likewise invaluable tools for studying cancer progression, scientists should start to consider novel ways around these adverse effects (e.g., the use or development of less immunogenic reporters). If growth differences are primarily attributable to cell arrest and this is being caused by luciferase or GFP expression, one potential way to mitigate this would be the use of inducible promoters whereby the reporters are not turned on until after the cells have arrested normally.

Alternatively, our initial transduction efficiency was quite low in this study, and potential clonal dominance needs to be considered. Previous work has shown that the bulk of a solid cancer mass is derived from a single cell rather than a variety of cells that proliferate at a similar rate to produce a heterogeneous tumour [15]. By selecting a relatively small subset of cells during the engineering process, it is possible that the resultant cell line was less brain trophic or less aggressive/metastatic than the initial cell line leading to significantly less brain tumour burden in these animals. Furthermore, our naïve 4T1BR5 cell line was truly “naïve,” and thus, we were unable to mock sort and expand them based on reporter gene expression as we did with our engineered cell line. Those using engineered cell lines for reporter gene imaging should provide a thorough explanation of how the cells were engineered to express those reporters. The vector used the selection process, and the purity of the cells will all provide information on how well the resultant cell line represents the initial population.

Previous studies have shown the types of genetic manipulations or variations in culture conditions that are necessary for reporter gene imaging have the potential to alter the cell's behavior both *in vitro* and *in vivo* [12]. Numerous groups have also shown engineered cells can have a similar growth rate to naïve cells *in vitro* but report a significantly slower growth rate *in vivo* compared to naïve cells [9, 12, 16], and that these differences can be related to the expression of a specific reporter. In a study by Tiffen et al., B16-F10 tumours expressing GFP-P2A-luc (luciferase) grew significantly slower than tumours formed expressing GFP only. Similarly, previous work has shown a link between the amount of reporter expression in the engineered cells and the magnitude of effect on growth. Brutkiewicz et al. found that a high level of luciferase expression can severely inhibit *in vivo* tumour growth while a low level of expression showed similar tumour growth to naïve cells [10]. However, vying for a lower level of reporter expression would limit the detectability of the cancer cells *in vivo*, which would be of great value in studies evaluating small numbers of cells arresting in downstream organs or those evaluating micrometastasis development. In contrast, other groups have found no significant differences in *in vivo* tumour growth following luciferase engineering [11, 12]. Previous work has also shown engineering cells to express luciferase can lead to increased survival of animals compared to animals receiving an injection of naïve cells, suggesting luciferase expression may render cells less aggressive/metastatic [9]. Many of these studies have attributed differences to the expression of luciferase itself and disregarded other variables in the engineering process that may affect *in vivo* growth such as how many cells from the initial population were engineered to avoid selecting a subpopulation with altered tropism or potential effects on where the reporter genes are integrated which may affect gene expression or other confounding variables.

## 5. Conclusions

In summary, this study describes the application of cellular and molecular imaging tools to characterize *in vivo* growth differences between naïve and engineered cell lines in a well-established mouse model of experimental breast cancer brain metastasis. By employing cellular MRI, we have demonstrated for the first time that cell engineering can have a significant effect on cell arrest in the brain. This indicates engineering cancer cells with reporter genes may alter their tropism towards particular organs, and care should be taken when engineering cells for reporter gene imaging of cancerous, and possibly noncancerous, cell populations.

## Data Availability

The data used to support the findings of this study are included within the article.

## Supplementary Materials

Supplementary Materials Supplementary Figure 1. In vitro characterization of cell line: bioluminescence imaging was performed to assess the functionality of the firefly luciferase gene in engineered 4T1BR5 cells (a). A clonogenic assay was performed to determine differences in the ability of each cell line to form colonies (b). Flow cytometry was used to determine differences in mean GFP fluorescence intensity over multiple passages (c).
