# Supplementary figures and images for: Cellular MRI Reveals Altered Brain Arrest of Genetically Engineered Metastatic Breast Cancer Cells
Source: Contrast Media Mol Imaging. 2019 Jan 8;2019:6501231. doi: 10.1155/2019/6501231 (PMC6348811; doi:10.1155/2019/6501231)

## Slide 1
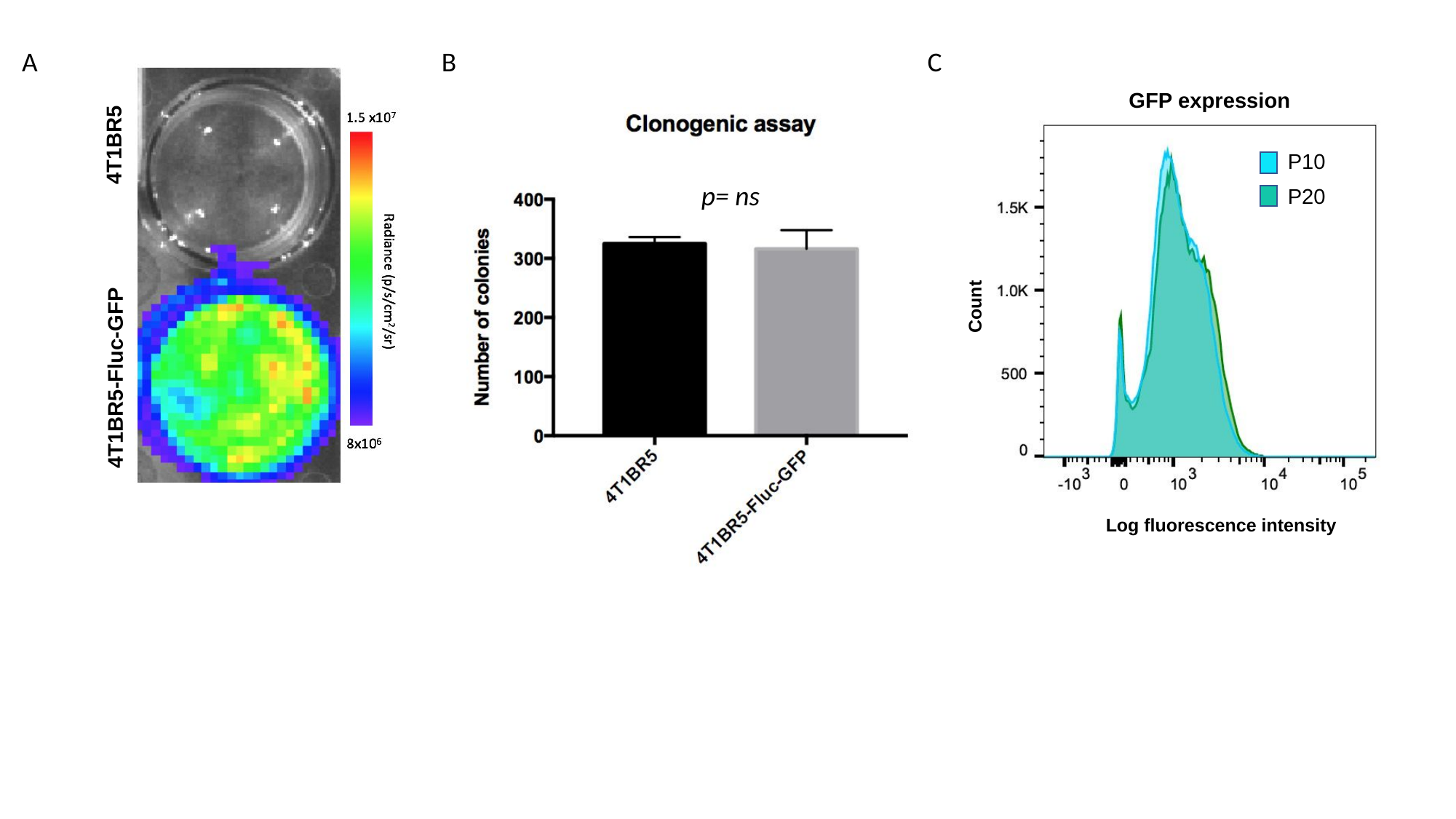

4T1BR5
A
B
C
GFP expression
P10
p= ns
P20
Count
4T1BR5-Fluc-GFP
Log fluorescence intensity

Supplement: Supplementary Materials — Supplementary Figure 1. In vitro characterization of cell line: bioluminescence imaging was performed to assess the functionality of the firefly luciferase gene in engineered 4T1BR5 cells (a). A clonogenic assay was performed to determine differences in the ability of each cell line to form colonies (b). Flow cytometry was used to determine differences in mean GFP fluorescence intensity over multiple passages (c). [file 6501231.f1.pptx]
